# The TNF-α/TNFR2 Pathway: Targeting a Brake to Release the Anti-tumor Immune Response

**DOI:** 10.3389/fcell.2021.725473

**Published:** 2021-10-12

**Authors:** Audrey Moatti, José L. Cohen

**Affiliations:** ^1^Université Paris-Est Créteil Val de Marne, INSERM, IMRB, Créteil, France; ^2^AP-HP, Groupe Hospitalo-Universitaire Chenevier Mondor, Centre d’Investigation Clinique Biothérapie, Créteil, France

**Keywords:** regulatory T cells (Tregs), immunotherapies, cancer, immunosuppression, anti-tumor action

## Abstract

Newly discovered anti-cancer immunotherapies, such as immune checkpoint inhibitors and chimeric antigen receptor T cells, focus on spurring the anti-tumor effector T cell (Teff) response. Although such strategies have already demonstrated a sustained beneficial effect in certain malignancies, a substantial proportion of treated patients does not respond. CD4^+^FOXP3^+^ regulatory T cells (Tregs), a suppressive subset of T cells, can impair anti-tumor responses and reduce the efficacy of currently available immunotherapies. An alternative view that has emerged over the last decade proposes to tackle this immune brake by targeting the suppressive action of Tregs on the anti-tumoral response. It was recently demonstrated that the tumor necrosis factor alpha (TNF-α) tumor necrosis factor receptor 2 (TNFR2) is critical for the phenotypic stabilization and suppressive function of human and mouse Tregs. The broad non-specific effects of TNF-α infusion in patients initially led clinicians to abandon this signaling pathway as first-line therapy against neoplasms. Previously unrecognized, TNFR2 has emerged recently as a legitimate target for anti-cancer immune checkpoint therapy. Considering the accumulation of pre-clinical data on the role of TNFR2 and clinical reports of TNFR2^+^ Tregs and tumor cells in cancer patients, it is now clear that a TNFR2-centered approach could be a viable strategy, once again making the TNF-α pathway a promising anti-cancer target. Here, we review the role of the TNFR2 signaling pathway in tolerance and the equilibrium of T cell responses and its connections with oncogenesis. We analyze recent discoveries concerning the targeting of TNFR2 in cancer, as well as the advantages, limitations, and perspectives of such a strategy.

## Introduction

The last few decades have marked an era of promising advances in the field of cancer therapy. The anti-tumor strategy landscape has shown several fundamental changes of paradigm, switching from cancer cell-centered approaches using chemo- and radiotherapy to strategies focused on the specific features of cells and the tumor microenvironment (TME), with targeted therapies, to therapies that enable the patient’s immune system to fulfill its role of destroying tumor cells. Recently approved anti-CD19-anti-CD3 bispecific monoclonal antibodies (mAb), immune checkpoint inhibitors (ICIs), and adoptive chimeric antigen receptor (CAR)-T cells were developed to enable patients’ effector T cells (Teffs) to better recognize and eradicate malignant cells (Waldman et al., 2020). These immunotherapeutic approaches have already demonstrated their efficacy against several malignancies with a poor prognosis, bringing hope to numerous patients.

To date, ICIs that have reached the patients’ bedside have focused on releasing effector CD8^+^ T cells from functional restrictions induced by the tumor and its micro-environment (Topalian et al., 2015; Fesnak et al., 2016; Khalil et al., 2016). However, physiologically, a minor subset of T cells oversees deployment of the adaptative response to protect tissue homeostasis, avoiding outrange proliferation of the Teff population. The crucial role of CD4 + FOXP3^+^ regulatory T cells (Tregs) in immune tolerance has led to a massive effort to better unveil their origin and suppressive function over the last 50 years (Sakaguchi et al., 2007). In the context of cancer, tumor and TME-associated cells often impair the balance between Teffs and Tregs, with an observed increase in the function or number of Tregs in several types of malignancies, as summarized in Table 1 (Curiel, 2008, p. 200). Although cytotoxic tumor-infiltrating lymphocytes (TILs) are often associated with favorable clinical outcomes, the relationship between FOXP3^+^ TIL and the prognosis is less clear. This controversy concerning the role of Tregs in malignancy may arise from various causes, including differences in the methods and markers used to identify Tregs between studies. Furthermore, the fact that the FOXP3^+^ population is not composed of solely suppressive cells (Tanaka and Sakaguchi, 2017) and that FOXP3 expression in neo-activated human Teff is transitory may also add a level of complexity to interpreting these studies (Saito et al., 2016).

**TABLE 1:**
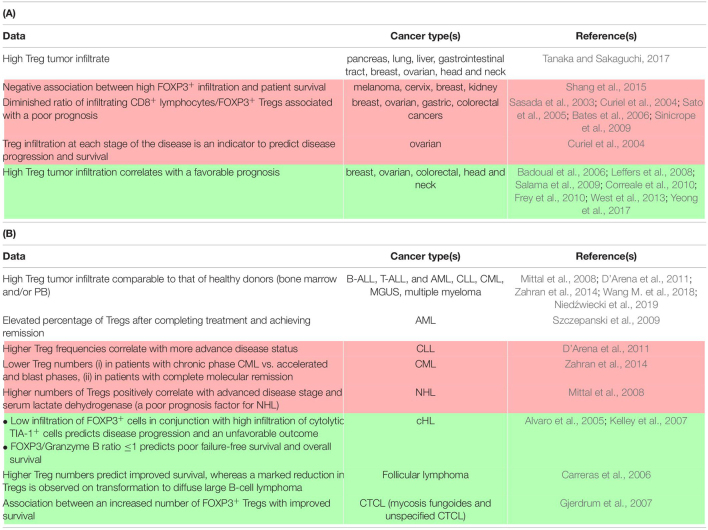
Detection and clinical association of Tregs in carcinomas (A) and hematological malignancies (B).

*White indicates data with no prognostic association, red a negative correlation between Treg infiltration and prognosis, and green a positive correlation. AML, acute myeloblastic leukemia; ALL, acute lymphoblastic leukemia; cHL, classical Hodgkin lymphoma; CML, chronic myeloid leukemia; CTCL, cutaneous T cell lymphoma; MGUS, monoclonal gammopathy of undetermined significance; NHL, non-Hodgkin lymphoma.*

Regardless of the mechanism, the inherent function of Tregs to suppress conventional T cell responses can be acutely detrimental for the immune control of cancer initiation, growth, and dissemination for several cancer types (Table 1). In addition, Tregs are likely to interfere with Teff-centered immunotherapies (Binnewies et al., 2018). Although these cells can serve as prognostic factors, it is also necessary to learn how to block this heterogeneous population in patients to overcome its immunosuppressive effects. Initially, mouse models allowed the demonstration of a beneficial effect of Treg depletion on the potency of the anti-tumor response using FOXP3^DTR^ mice or antibodies targeting highly expressed Treg receptors (Klages et al., 2010). Based on these observations, a myriad of potential therapeutic approaches directly targeting Tregs have been described in animal models and several are currently being assessed in clinical trials. In addition, several therapies already in use have a documented, yet still controversial, effect in impairing Tregs, either immunotherapies, such as ipilimumab [anti-cytotoxic T-lymphocyte antigen 4 (CTLA-4)] (Tang et al., 2018, p. 4), or certain chemotherapeutic agents, such as cyclophosphamide and fludarabine (Beyer et al., 2005; Walter et al., 2012). Specific Treg inhibition can refer to the blockade/impairment of: (i) thymic-derived Treg migration to the tumor site or, alternatively, the capacity of naïve T cells to become Tregs on site, (ii) their activation, proliferation, and/or survival, and (iii) their suppressive function. Studies have exploited the chemokine network (mainly the CCR4/CCL22 axis (Ishida et al., 2012; Ni et al., 2015), the IL-2/CD25 pathway (Ross and Cantrell, 2018), and co-stimulatory molecules, such as GITR (Killock, 2019; Zappasodi et al., 2019), inducible T cell costimulatory (ICOS) (Le et al., 2016; Mo et al., 2017; Yap et al., 2018), and OX40 (Aspeslagh et al., 2016).

Despite these emerging approaches to block or deplete Tregs, they still show limitations because of the lack of Treg-specific biomarkers and the potential induction of autoimmunity as a consequence of systemic Treg impairment (Stephens et al., 2001; Kim J. et al., 2009). Additionally, Treg depletion may be followed by their rapid reconstitution, potentially resulting in a higher Treg frequency than their level prior to depletion (Mahnke et al., 2007; Berod et al., 2014). Therefore, developing new tools to circumvent tolerance toward malignant cells is still a priority. In this respect, the type II receptor of tumor necrosis factor alpha (TNF-α) (TNFR2) represents another hope in targeting Tregs, including in the tumoral context (Cohen and Wood, 2017). Due to its particular pattern of expression by cells of the immune system, preferentially immunosuppressive cells, targeting TNFR2 could permit the tuned modulation of both innate and adaptive responses in diverse pathogenic contexts (Salomon et al., 2018; Wajant and Beilhack, 2019). After discussing the role of the TNF-α/TNFR2 in tolerance, we then present and discuss the most recent developments that have led to the consideration of TNFR2 as a new brake to impede the anti-tumor immune response through its beneficial role on Tregs and the associated therapeutic perspectives for this molecule as a novel target for cancer immunotherapy.

## The Critical Function of TNF-α/TNFR2 in Tolerance

### Comprehensive Overview of the TNF-α Signaling Pathway

#### Pattern of Expression of TNF-α and Its Receptors

Tumor necrosis factor alpha is currently considered to be one of the most pleiotropic cytokines described in mammals, with roles spanning virtually every biological system beyond its activity in immune system physiology. Indeed, this transmembrane protein is not only expressed by immune cells, such as monocytes/macrophages (including microglia in the nervous system), B cells, activated T and NK cells, but also by a diverse array of non-immune cells, such as fibroblasts, keratinocytes, astrocytes, endothelial cells, epithelial cells, and many cancer cells (Sedger and McDermott, 2014). Transmembrane TNF-α (tmTNF-α) assembles into a homotrimer that is cleaved by the matrix metalloprotease TNF-α-converting enzyme (TACE/ADAM17) releasing a soluble form of the TNF-α (sTNF-α) homotrimer, responsible for the endocrine function of TNF-α (Kriegler et al., 1988; Black et al., 1997; Moss et al., 1997).

Both forms can bind to structurally related but functionally distinct receptors: TNFR1 (p55/60), which binds to tmTNF-α, as well as sTNF-α, and TNFR2 (p75/80), which shows higher affinity and is more robustly activated by tmTNF-α than sTNF-α (Grell et al., 1995). TNFR1 is ubiquitously expressed in almost any cell type at a low level, whereas TNFR2 expression is finely regulated and limited to several cell types of the immune system, including CD4^+^ and CD8^+^ T cells, but also plays an important role in cells of the vasculature and muscle and brain tissues (Faustman and Davis, 2010; Fischer et al., 2011; Puimège et al., 2014; Pegoretti et al., 2018). A high density of TNFR1 and TNFR2 has been observed on myeloid cells (monocytes, macrophages, and dendritic cells), in which both pathways are interconnected, promoting their activation, proliferation, and survival (Rossol et al., 2007; Maney et al., 2014; Wajant and Siegmund, 2019). Myeloid-derived suppressor cells (MDSCs), an inflammation-induced population, appears to be the one myeloid population that specifically requires TNFR2 for its induction and suppressive functions (Zhao et al., 2012; Hu et al., 2014; Polz et al., 2014; Ham et al., 2015). Finally, the TNF-α/TNFR2 signaling pathway has very recently been shown to be a key regulatory factor for the immunosuppressive effect of mesenchymal stem cells and neural and endothelial progenitor cells (Beldi et al., 2020; Naserian et al., 2020; Shamdani et al., 2020). Overall, these observations support the existence of a TNFR2-dependant network of immunosuppressive cells that have been ignored until recently, that could help broadening the therapeutic landscape.

#### TNF-α/TNFR2 Signaling Pathways

**FIGURE 1:**
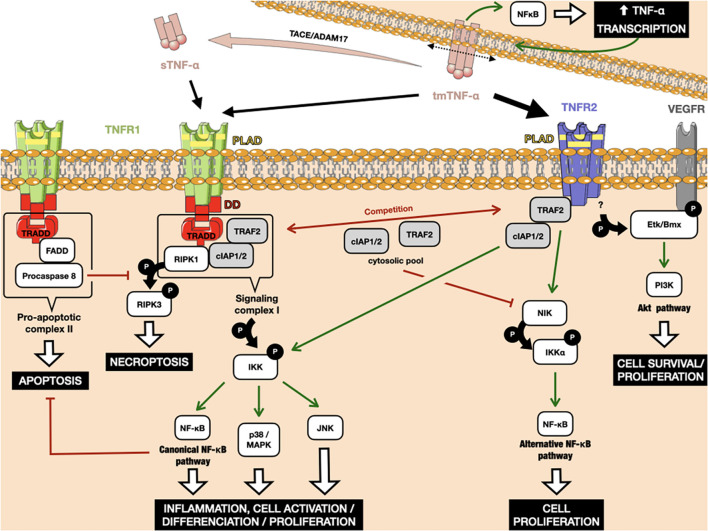
Interplay between the TNFR1 and TNFR2 downstream signaling pathways. TNFR1 can bind to tm-TNF-α, as well as its soluble form after cleavage by the metalloproteinase TACE/ADAM17. TNFR2 binds to tm-TNF-α with higher affinity. Binding of Tm-TNF-α to TNFRs induces a positive retro-control loop on its transcription. TNFR1 activation can lead to apoptosis and necroptosis through interaction between its death domains and the TRADD adaptor. Both TNFR1 and TNFR2 can recruit cIAP1/2, leading to the activation of classical NF-κB. TRAF2 weakly binds to TNFR2, leading to activation of the alternative NF-κB pathway. The amount of cIAP1/2 and TRAF2 in the cytosol is crucial in cells that simultaneously express TNFR1 and TNFR2, as the consumption of these molecular actors after TNFR2 activation leads to the preferential activation of death pathways under TNFR1 rather than those of proliferation and survival. Red arrows indicate inhibition. Green arrows indicate activation. P: phosphorylation.

Tumor necrosis factor receptor 1 and TNFR2 share similar extracellular TNF-α-binding motifs, including the membrane-distant pre-ligand binding assembly domain (PLAD), which is important for the ligand-mediated formation of active receptor complexes (Chan et al., 2000). As both receptors lack intrinsic enzyme activity, they need to recruit cytosolic actors upon ligand binding to initiate intracellular signal transduction. The two TNF-α receptors differ highly in their intracellular structure, which is responsible for their divergent activity. Simply put, TNFR1 belongs to the family of death domain-containing receptors and is responsible for cell death, whereas TNFR2 is a TNFR-associated factor (TRAF)-interacting receptor, without a death domain, that favors cell activation (Wajant et al., 2003). However, the reality is much more complex, with the addition of the interplay between the intracellular pathways of the two receptors reflecting the broad range of biological actions of TNF-α. The signaling pathways are discussed in detail below and presented in Figure 1.

The TNFR1 death domain preferentially interacts with an adaptor protein, called TNFR1-associated death-domain (TRADD) protein (Dempsey et al., 2003). TRADD further recruits another two adaptor proteins, receptor-interacting protein kinase 1 (RIPK1) and TRAF2, thus forming an enzymatic complex signalosome, also known as signaling complex I. One of the main targets of complex I is the enzyme complex IκB kinase (IKK). Phosphorylation of IKK leads, in turn, to the canonical activation of nuclear factor-κB (NF-κB), as well as members of the family of MAP kinases, such as c-Jun kinase (JNK) and p38 MAPK (Natoli et al., 1997; Brinkman et al., 1999). The respective target genes of these proteins allow the organism to respond effectively to environmental changes. The classical NF-κB pathway is activated by a variety of inflammatory signals, resulting in coordinate expression of multiple inflammatory and innate immune genes (Liu et al., 2017). The JNK pathway and p38 signaling are strongly activated by surrounding stress and inflammatory cytokines. They contribute to a broad range of reactions, including inflammation, apoptosis, cell activation/migration/differentiation, cell cycle regulation, cytokine production, and metabolism (Morrison, 2012). Alternatively, signaling complex I can also be internalized and then converted to a death-inducing signaling complex, so-called pro-apoptotic complex II, by adaptor protein Fas-associated protein with death domain (FADD). Complex II includes procaspase 8, which is activated by autocatalytic cleavage, initiating downstream caspase cascades and ultimately inducing cell apoptosis (Micheau and Tschopp, 2003; Schneider-Brachert et al., 2004). If caspase 8 is absent or inactivated, kinase-active RIPK1 recruits and activates RIPK3, resulting in the formation of the necrosome and further execution of necroptosis via membrane permeabilization (Vanden Berghe et al., 2014; Grootjans et al., 2017). Caspases and their inhibitors are pivotal elements in deciding the cell’s fate after TNF-α/TNFR1 triggering, along with the engagement of NF-κB-mediated anti-apoptotic signaling pathways, which are able to delay the time of death (Schliemann et al., 2011).

In contrast to the well-characterized TNFR1 signaling pathways and their physiological relevance, TNFR2-mediated signaling pathways were uncovered much later. Because of the lack of a death domain, TNFR2 is unable to recruit TRADD protein, but can instead weakly bind to TRAF2 directly (Rothe et al., 1995, p. 2). Under these conditions, TRAF2 induces the non-canonical NF-κB pathway, through the activation of NF-κB-inducing kinase (NIK), which further leads to the phosphorylation of IKKα and the processing of p100, a crucial step in the nuclear translocation of p52/RelB (Borghi et al., 2018). The alternative NF-κB pathway acts quite distinctly from the classical one, for example by being dispensable for the initial activation of naive T cells through TCR signaling but crucial for the *in vivo* generation and maintenance of effector and memory T cells (Sun, 2017). However, upon binding to TRAF2, TNFR2 can also recruit cIAP1/2 proteins, which are involved in TNFR1-mediated NF-κB activation, creating crosstalk between the TNFR pathways. TNFR2 pathway activation consumes the cytosolic pool of the TRAF2-cIAP1/2 complex, limiting its availability for other receptors, including TNFR1. Due to the role of TRAF2 and cIAPs in preventing apoptosis and necroptosis in the context of TNFR1 signaling, TNFR2-mediated deprivation of these molecules is able to enhance TNFR1-induced cell death in macrophages (Siegmund et al., 2016). Finally, another notable adaptor protein, called endothelial/epithelial protein tyrosine kinase (Etk/BMX), has been described that can interact with TNFR2 in the absence of ligand (Pan et al., 2002). TNFR2-mediated Etk phosphorylation is able to partially activate the growth factor receptor VEGFR2, resulting in cell survival and proliferation through PI3K/Akt activation (Fischer et al., 2015, p. 1; Ortí-Casañ et al., 2019).

An amplification loop has been reported for the TNF-α pathway (Zuckerman et al., 1991). TNF-α production is highly inducible, up to 10,000 fold, due to transcriptional upregulation of mRNA production by, among others, the NF-κB transcription factor (Varfolomeev and Vucic, 2018). In addition, TNF-α expression depends on p38-MAPK and JNK signaling at the post-transcriptional level, which act by modulating mRNA stability and translational efficiency (Falvo et al., 2010). As TNF-α binding to the TNFR activates the MAP kinase signaling cascades and transcription factors of the NF-κB family, it induces its own transcription in cells that express its receptors (Wajant and Beilhack, 2019). Furthermore, TNFR-binding can induce reverse signaling in tmTNF-α producing cells (Tartaglia et al., 1993). Such signal transduction results in the activation of NF-κB, this time within the TNF-α-producing cell, leading again to greater TNF-α transcription (Watts et al., 1999; Xin et al., 2006).

### TNF-α/TNFR2 in the Control of the T Cell Response

#### Conventional T Cells

Most described TNF-α-mediated proinflammatory functions are mediated via TNFR1 expression in a multitude of immune cells, including T cells (Mehta et al., 2018). TNFR2 is also found on activated conventional T cells, in which it acts as a co-stimulatory molecule in a unique non-redundant manner, similarly to CD28 co-stimulation (Aspalter et al., 2003; Reiner, 2007). TNFR2 deficiency in knockout mice impairs the proliferative ability of conventional CD4^+^ and CD8^+^ cells and decreases their production of TNF-α, IFN-γ, and IL-2 in response to TCR stimulation (Kim and Teh, 2001; Kim et al., 2006, 2008). Intriguingly, TNF-α produced by activated Teffs under inflammatory conditions has been shown to be necessary for the stimulation of Treg expansion and the enhancement of their suppressive function (Chen et al., 2007; Grinberg-Bleyer et al., 2010; Leclerc et al., 2016). TNFR2 is not only essential for optimal Teff proliferation and activation but also the induction of activation-induced cell death (AICD), which terminates the proliferative response, a process that is dependent on the TNFR2 downstream actor TRAF2 (Twu et al., 2011). Consistent with this role of TNFR2, TNFR2^–/–^ CD8^+^ T lymphocytes show high resistance to AICD, leading to the worsening of colonic inflammation (Punit et al., 2015). Thus, TNFR2 signaling is responsible for dual effects in T cells, as its activation stimulates establishment of the effector response while permitting its subsequent regulation through (i) the death of effector cells and (ii) the stimulation of the Treg subpopulation, which also helps to terminate the immune reaction.

#### Regulatory T Cells

Although TNFR1 expression does not differ between Tregs and other T cells, human Tregs constitutively express high levels of TNFR2 relative to conventional T cells in the steady-state (Fischer et al., 2020). Hence, the use of a target-agnostic phage display screening approach on human Tregs to find antibodies that preferentially bind to them, rather than Teffs, has surprisingly led to only finding candidates that specifically bind to TNFR2 (Williams et al., 2016). Thus, in addition to being a Treg marker, several studies conducted by Chen et al. (2008, 2010a, 2010b) showed that TNFR2 expression by Tregs has important functional implications and defines a maximally suppressive subset of mouse and human Tregs (Chen and Oppenheim, 2011b, c). In particular, it was shown that the level of expression of TNFR2 correlates with the suppressive potential of nTregs (Chen et al., 2007), indicating that the most potent suppressors are highly susceptible to TNFR2 activation. These findings have been confirmed by many other groups since these pioneering findings (Ablamunits et al., 2010; Grinberg-Bleyer et al., 2010; Kleijwegt et al., 2010; Housley et al., 2011; Okubo et al., 2013; Atretkhany et al., 2018). Although the membrane-bound form of TNFR2 can be either immunosuppressive or immunostimulatory, depending on which cell type expresses it, the function of the soluble form of TNFR2 appears to be consistently immunosuppressive (van Mierlo et al., 2008). Activated Tregs can release high amounts of sTNFR2, which, at least partially, represents an additional immunosuppressive mechanism of Tregs. Interestingly, it appears that these features are not restricted to CD4^+^ Tregs, as the most potent CD8^+^ suppressors are also characterized by the expression of TNFR2, although exploratory studies are required to confirm such results about this unrecognized regulatory subset (Ablamunits et al., 2010; Horwitz et al., 2013).

It is now well recognized that TNFR2 also contributes to the expansion of nTregs *in vitro* and *in vivo* (Chen et al., 2007, 2008; Okubo et al., 2013; Chopra et al., 2016; Fischer et al., 2017, 2018, 2019a, b; Padutsch et al., 2019). Recently, TNFR2 ligation was confirmed to enhance cell proliferation through the non-canonical NF-κB pathway in human Tregs, enhancing IL-2-induced cell proliferation (Wang J. et al., 2018). However, in mouse Tregs, the activation of p38 MAPK via the classic NF-κB signal appears to also be important for TNFR2-induced proliferation (He et al., 2018). Much information about the role of TNFR2 in Treg expansion and phenotypic stability has also come from the field of adoptive Treg cell therapy, used to aid tolerance in the context of autoimmunity, organ rejection, and GvHD. A non-commercial agonistic TNFR2 antibody in standard *ex vivo* Treg expansion protocols was shown to confer improved suppressive activity while reducing Treg heterogeneity (Okubo et al., 2013). Furthermore, using the TNFR2-specific mAb MR2-1 as an agonist, TNFR2 signaling promoted the expansion of low-purity MACS-isolated Treg preparations to homogenous Treg populations, stable under further restimulation (He et al., 2016). These studies, in accordance with the above-mentioned *in vitro* and *in vivo* data, demonstrate the role of TNFR2 in the suppressive potency of Tregs and encourage its utilization to improve *ex vivo* Treg expansion methods for clinical applications.

#### In Autoimmune Settings

Given the above-mentioned function of TNFR2 in Tregs and conventional T cell responses, these observations raise the question of the role of TNFR2 in the interplay between inflammatory and regulatory pathways in human immune pathologies. Initially, data about TNFR2 role in tolerance has come from the field of autoimmune diseases. Interestingly, TNFR2 polymorphisms can be found in patients with several inflammatory and auto-immune diseases, cementing its pivotal link with human immune tolerance (Pierik et al., 2004; Oregón-Romero et al., 2006; Horiuchi et al., 2007; Song et al., 2014). A role for TNF-α in suppressing systemic autoimmune responses has been emphasized by anti-TNF-α therapies for rheumatoid arthritis and inflammatory bowel disease patients. Drug-induced systemic lupus erythematosus symptoms were reported for certain patients after treatment with anti-TNF-α mAbs and soluble TNF-α receptors (Sandborn and Hanauer, 1999; Kollias and Kontoyiannis, 2002; Shakoor et al., 2002).

Although the efficacy of anti-TNF-α treatment requires no further demonstration in autoimmune diseases, it is still a relatively non-specific approach that does not affect solely autoreactive actors. Indeed, as implied by its name, TNF-α inhibition can result in rare but severe occurrences of treatment-induced hematological malignancies (Hansel et al., 2010). The perceived increase in such hematological malignancies has led the World Health Organization classification of tumors to include the category “iatrogenic immunodeficiency-associated lymphoproliferative disease,” and the risk of virally transformed tumors is also being closely watched (Campo et al., 2011). Although the risk of anti-TNF-α-induced cancer for autoimmune patients is still debated, it provides a good example of a situation in which promoting tolerance via TNFR2 targeting would presumably be a better strategy than targeting TNF-α. The incredibly wide range of physiological functions dependent on TNF-α/TNFR1 in all body systems, including cancer immunosurveillance, render targeting this pathway highly challenging (Faustman and Davis, 2010, 2013).

## The Role of TNF-α/TNFR2 in Malignancy

### In the Tumor Microenvironment

Although immunosuppressive cells do not have a monopoly on TNFR2 expression in the steady-state, a large number of studies suggest that TNFR2^+^ Tregs with high suppressive capacity are strongly represented in the TME of cancer patients and sometimes the peripheral blood, comparable to findings in mouse models (Chen et al., 2008). The first remarkable observation concerning TNFR2 in the context of cancer was the decrease in tumor growth and metastasis described in TNFR2 knockout mice, despite the TNFR2 costimulatory function on Teffs (Sasi et al., 2012; Chopra et al., 2013; Ham et al., 2015). TNFR2−deficient mice show reduced infiltration and induction of MDSCs, coinciding with a diminution in the number of Tregs inside the tumors, confirming the requirement of TNFR2 for the participation of these immunosuppressive cells in the TME (Zhao et al., 2012). Furthermore, although the beneficial effects of TNF-α on CD8^+^ Teffs are mainly mediated through TNFR2 (Zheng et al., 1995; Calzascia et al., 2007), Chen et al. (2010a) showed that the upregulation of TNFR2 on intratumoral Tregs enables them to overcome the greater resistance to suppression of conventional intratumoral TNFR2^+^ T cells. Overall, these data support the idea of a diverted role of TNFR2 in tumors in favor of a predominant immunosuppressive TME, notwithstanding the relevance of its function in T cells activation mentioned earlier.

In cancer patients, there is now compelling evidence that TNFR2^+^ Tregs accumulate in TILs in Sézary Syndrome and cervical cancer and tumor ascites in ovarian cancer. This population is also elevated in the peripheral blood of acute myeloblastic leukemia (AML) patients, as well as that of hepatocellular carcinoma, lung, and cervical cancer patients (He et al., 2019). Remarkably, in the peripheral blood samples of lung cancer patients, the expression of TNFR2 on Tregs appears to correlate better with FOXP3 expression than the CD25^+^CD127^–^ combination (Yan et al., 2015, p. 2). Additionally, TNFR2^+^ Tregs were associated with lymphatic invasion, distant metastases, and a more advanced clinical stage of lung cancer. This and other observation highlight the pivotal role of TNFR2 expression in Tregs in the context of human cancer, consistent with the conclusions based on mouse models. An updated list of the studies available on TNFR2^+^ Tregs in mice and humans, and their outlines are provided in Table 2. Interestingly, Tregs from the CD8^+^ T cell subset have been recently found to also constitutively express TNFR2 (Ablamunits et al., 2010; Horwitz et al., 2013). These cells, less studied than their CD4^+^ counterpart, have also been implicated in the maintenance of pro-tumoral tolerance (Zhang et al., 2018).

**TABLE 2:** Role of TNFR2^+^ Tregs in cancer immunology.

**(A)**			

Tumor type	Mice		Reference(s)
Experimental metastasis	• In TNFR2^–/–^ mice with colon (MC-38) or lung (H-59) carcinoma, metastasis and Tregs accumulation are reduced • In WT mice, treatment with TNFR2 antisense oligodeoxynucleotides inhibits hepatic metastasis • In mice with TNFα- or TNFR2 immune cell-restricted deficiency, melanoma (B16F10-Luc) metastasis to the lung and numbers of Treg infiltration in lungs are decreased		Chopra et al., 2013; Ham et al., 2015

**(B)**			

**Tumor type**	**Mice**	**Patients**	**Reference(s)**

Lung cancer	• The proportion of TNFR2^+^ cells in CD4^+^CD25^+^ TILs is >70%, higher than in peripheral lymphoid organs	• Increased proportion of TNFR2^+^ Tregs in PB (>TNFR2^+^ Teff) • TNFR2^+^ Tregs positively correlate with lymphatic invasion, distant metastasis, and clinical stage • TNFR2^+^ Tregs are more proliferative, active, and suppressive	Chen et al., 2008; Yan et al., 2015, p. 2
Hepatocellular carcinoma and colon cancer	• CD103^+^ Tregs express higher levels of TNFR2 than CD103- Tregs in the spleen and tumor (CT26 and BNL cell model) • Blockade by an anti-TNFR2 mAb or by a soluble TNFR2 fusion protein (sTNFR2-Fc) inhibits TNF-α-induced expansion of CD103^+^ Tregs *in vitro* • Blockade by sTNFR2-Fc after cyclophosphamide treatment • inhibits tumor growth	• Increased proportion of CD45RA^–^ FOXP3^hi^ effector Tregs in PB, with high expression of CTLA-4, CCR5, and TNFR2	Chang et al., 2015
Colon cancer	• TNFR2-blocking Ab combined with CpG ODN (TLR9 agonist) reduces the proportion of TIL TNFR2^+^ Tregs, increases cytotoxic IFN-γ^+^ CD8^+^ TILs, and inhibits CT26 growth	• Higher Treg number in colon cancer tissues than in surrounding unaffected mucosa	Williams et al., 2016; Nie et al., 2018

**(C)**			

**Tumor type**	**Patients**		**Reference(s)**

Ovarian cancer	• Higher proportion of CD4^+^CD25^hi^TNFR2^+^ Tregs in tumor ascites • Up-regulation of CD39, CD73, GARP, and TGF-β in this subpopulation • TNFR2^+^ Tregs dampen local IFN-γ and IL-2 production by Teff (more than blood Tregs)		Govindaraj et al., 2013a; Torrey et al., 2017
Cervical cancer	• PB and TNFR2^+^Treg TILs significantly elevated in patients with cervical intraepithelial neoplasia and cancer • Circulating s-TNFR1 and s-TNFR2 elevated in cervical cancer patients • Percentage of peripheral TNFR2^+^Tregs inversely correlates with the clinical stage of cervical cancer		Zhang et al., 2017
AML	• In newly diagnosed patients vs. those in complete remission and healthy controls: (i) higher production of TNF-α by CD4^+^ T cells (mostly Th17), (ii) higher circulating frequencies of CD4^+^CD25^+/hi^ Tregs, (iii) higher TNFR2 expression on Tregs, preferentially on CD4^+^CD25^hi^ Tregs • Most of the patients’ Tregs express TNFR2^+^, have a high migration potential toward the bone marrow, up-regulate CTLA-4 and CD73, and produce more IL-10 and TGF-β. • TNFR2^+^ Tregs in the PB and bone marrow are selectively decreased after epigenetic therapy with panobinostat (histone deacetylase inhibitor) and azacytidine (demethylating agent) in responder patients in association with a beneficial clinical response • This treatment led to a reduction in TNFR2^+^ Tregs associated with their down-regulation of FOXP3 and CTLA-4 expression and an increase in IFN-γ and IL-2 production by Teff within the bone marrow		Govindaraj et al., 2014b; Wang M. et al., 2018
Sézary syndrome	• High expression of TNFR2 on Tregs and cancer cells • TNFR2 antagonist killed tumor cells, restored the CD26^–^ subpopulation, and reduced the number of Tregs and ratio of Tregs/Teff		Torrey et al., 2018

*Data found in mouse models (A), both mice and patients (B), or restricted to cancer patients (C). WT: wildtype.*

Concerning TNFR2^+^ myeloid cells, the presence of MDSCs has been noted in plasmacytoma, fibrosarcoma, and liver and lung cancer (Sheng et al., 2018). Additionally, the presence of tumor-associated macrophages expressing TNFR2 has been shown to correlate with malignancy grade and metastasis in human triple-negative breast cancer (Frankenberger et al., 2015). Lastly, TNFR2 expression on endothelial cells makes it essential for tumor angiogenesis. In highly vascularized murine lung tumor xenografts tumor growth was inhibited in TNFR2^–/–^ mice, in correlation with decreases in VEGF expression and capillary density, as well as bone marrow-derived endothelial progenitor cell incorporation into the functional capillary network (Sasi et al., 2012).

### In Cancer Cells

Apart from its benefits concerning tolerance, another crucial aspect concerning the role of the TNFR2 pathway in improving carcinogenesis relies on its oncogenic features. Indeed, TNFR2-dependant NF-κB activation in epithelial cells induces carcinogenesis and the absence of this mechanism may have taken part as well in the observed impairment of tumor growth in TNFR2^–/–^ mice (Onizawa et al., 2009; Suzuki et al., 2014; Nagaishi et al., 2016). Aberrant expression of TNFR2 on tumor cells has been reported in human hematological malignancies, including Hodgkin lymphoma, cutaneous T cell lymphoma (CTCL), and multiple myeloma, as well as in breast, skin, ovarian, colon, and renal cell cancers (Arnott et al., 2004; Uhlén et al., 2005; Hamilton et al., 2011; Rauert et al., 2011; Nakayama et al., 2014; Ungewickell et al., 2015; Al-Lamki et al., 2016; Williams et al., 2016; Yang et al., 2018). For example, point mutations and genomic gains of the TNFR2 gene (*TNFRSF1B*) enhancing the activation of the non-canonical NF-κB signaling pathway have been described in 18% of patients with recurrent CTCL, for both mycosis fungoides and Sézary syndrome (Ungewickell et al., 2015). Remarkably, recombinant human TNF-α has been shown to increase the quantity of TNFR2 expressed at the surface for a number of tumor epithelial-like cell lines (Medvedev et al., 1996; Alshevskaya et al., 2020). Additionally, as mentioned earlier, the binding of tmTNF-α to TNFR2 can induce reverse signaling, inducing survival via the NF-κB pathway in lymphoma cells, all the more so with soluble TNFR2 which is highly secreted by Tregs in the TME (Zhang et al., 2008). In turn, it appears that TNFR2 overexpression by cancer cells in a model of colon carcinoma (CT26) is associated with a greater presence of TNFR2^+^ Tregs in draining lymph nodes and four time more sTNFR2 in the peripheral blood (Chen et al., 2018). In colorectal cancer patients, higher sTNFR2 levels are associated with a significant increase in overall mortality (Babic et al., 2016). Thus, TNFR2 is directly involved in uncontrolled tumor expansion, a feature that supplements its previously described role in maintaining an immunosuppressive milieu around malignant cells.

## Specific TNFR2-Targeting in Cancer

### The Rationale Behind TNFR2 Blockade

Although the qualification of “tumor necrosis” has withstood the test of time, the reality is less straightforward. Due to its plethora of functions through its two receptors, TNF-α is responsible for divergent actions in the context of cancer (Montfort et al., 2019). Although its function through TNFR1 effectively favors the death of cancer cells while promoting the T cell pro-inflammatory response via NF-κB signaling, the activation of TNFR2 on immunosuppressive cells recruited by the tumor could be detrimental for anti-cancer responses. Considering this refined view of TNF-α functions, several studies have focused on sensitizing cancer cells to TNFR1-induced apoptosis, for example by inhibiting survival signals, such as NF-κB, in combined therapy with TNF-α (Wang and Lin, 2008). However, these approaches are, as the historical attempts to use recombinant TNF-α in cancer patients, not specific to cancer cells, with a high risk of off-target effects (Roberts et al., 2011). A more specific approach would be to block TNFR2 to focus the therapy on the immunosuppressive cells that accumulate during carcinogenesis, hoping to:

(i) eliminate the detrimental immunosuppressive TME, including infiltrated TNFR2^+^ Tregs, to consequently awaken the anti-tumor response,
(ii) while redirecting TNF-α to TNFR1 expressed at the surface of immune effector cells, promoting an inflammatory response.

Furthermore, many tumors appear to start expressing TNFR2 during their transformation or originate from cells that express it in the case of immune cell-derived neoplasms, offering a chance to directly impair tumor evolution by blocking an oncogene.

### Pioneering Approaches for TNFR2 Blockade

Based on these compelling assertions, several studies have addressed the feasibility of therapeutic TNFR2 blockade in animal cancer models (Table 2.). The group of Faustman has developed two dominant human TNFR2 antagonist mAbs that lock TNFR2 in the form of antiparallel dimmers, preventing further TNF-α binding (Torrey et al., 2017). These compounds were able to kill the patients’ Tregs isolated from ovarian cancer ascites more potently than those from healthy donors, supposedly due to the high TNFR2 expression on TME-infiltrating Tregs. Thus, these antagonists can preferentially suppress tumor-associated Treg activity with no or only a minor inhibitory effect on regular Tregs in the periphery, permitting the maintenance of immunological homeostasis. On the other hand, TNFR2 antagonistic mAbs were able to directly kill TNFR2-expressing ovarian cancer cell lines *in vitro*. This last observation fosters the hypothesis of the synergistic action of TNFR2 on Tregs and malignant cells. Importantly, tumor antigens released from dead cancer cells can promote quiescent antitumor immune responses, triggered, in the meantime, by the attenuation of Treg activity. Similar observations were made by the same group in another *in vitro* study in which cancer cells and lymphocytes were isolated from patients with end-stage Sézary syndrome, an interesting scenario for TNFR2 blockade, because, as already mentioned, a portion of these malignant cells show Treg features (Torrey et al., 2018).

TNFR2^+^ Treg depletion augments the efficacy of chemotherapy in pre-clinical studies (van der Most et al., 2009). In a mouse model, the use of the alkylating agent cyclophosphamide depleted TNFR2^+^ Tregs by inducing cell death of replicating Tregs co-expressing TNFR2 and the cellular proliferation marker KI-67. However, the re-expansion of Tregs from lymphodepletion can suppress the effective antitumor immunity developed after cyclophosphamide treatment. Interestingly, TNF-α blockade by etanercept inhibits TNFR2^+^ Treg expansion during recovery from cyclophosphamide-induced lymphodepletion and markedly inhibits the growth of established CT26 tumors in mice, without affecting CD8^+^ T cell activation (Chang et al., 2015). In the same colon cancer model, as well as in 4T1 breast cancers, the combination of a TNFR2-blocking mAb with an immune stimulator (toll-like receptor agonist) markedly enhanced the antitumor efficacy of immunotherapy by reducing the number of tumor-infiltrating TNFR2^+^ Tregs and increasing the number of IFN-γ-producing CD8^+^ cells (Nie et al., 2018). Notably, the antagonistic TNFR2 antibody TR75-54.7 inhibited the growth of mammary carcinoma more efficiently than a CD25 antagonist. In addition, certain pharmacological agents regulate the expression of TNF-α and/or of its receptors. For example, thalidomide and its analogs prevent the surface expression of TNFR2 on activated T cells, which is associated with the inhibition of TNFR2 protein trafficking to the cell membrane (Marriott et al., 2002). Treating AML patients with azacitidine and lenalidomide, a thalidomide derivative, can reduce TNFR2 expression on T cells, as well as the number of TNFR2^+^ Tregs, *in vivo*, leading to enhanced effector immune function (Govindaraj et al., 2014a).

### Impact of TNFR2 Blockade on the Effector T Cell/Regulatory T Cell Equilibrium

**FIGURE 2:**
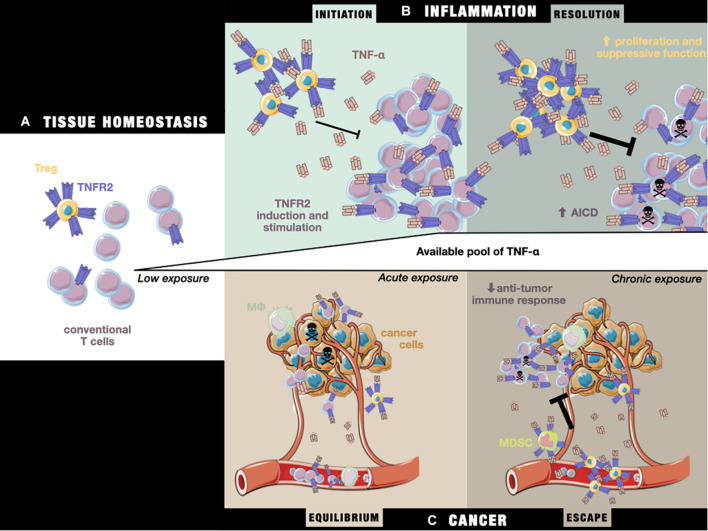
The impact of TNF-α/TNFRs on the T cell equilibrium during **(A)** tissue homeostasis, **(B)** the initiation and resolution of inflammation, and **(C)** cancer equilibrium and escape. **(A)** In the steady state, TNFR2 is more highly expressed by Tregs than Teffs. **(B)** Under inflammatory conditions, exposure to initiation-phase cytokines (including TNF-α) induces higher expression of TNFR2 on T cells and co-stimulation signaling, aiding the T cell response. Tregs are unable to fully repress the Teff response. Later, during the resolution phase, chronic exposure to TNF-α sensitizes Teffs to cell death through TNFR2 activation, while Tregs are able to more potently inhibit Teffs by the stabilization and stimulation of their suppressive functions, leading to restoration of the steady state situation described in panel **(A)**. **(C)** During tumor growth, tumor cells and the TME produce TNF-α, leading to a T cell response against tumor cells by peripheral Teffs that have been attracted to the tumor site. In a state of equilibrium, this allows the elimination of a pool of cancer cells, while others more resistant to the immune response continue to proliferate. Over time, the TME evolves and attracts an increasing number of immunosuppressive TNFR2^+^ cells (including Tregs and MDSCs), leading to suppression of the anti-tumor response, while the chronic exposure of Teffs to TNF-α drives them toward AICD, unleashing tumor growth and allowing tumor cells to escape from immune control.

A crucial point concerning the pattern of TNFR2 expression is its change under inflammation. Similarly to CD25, conventional T cells upregulate TNFR2 upon TCR activation (Govindaraj et al., 2013b). This raises the question of a potentially deleterious effect on the adaptive response after TNFR2 blockade and, more specifically, on the cytotoxic anti-tumor CD8^+^ response. To address this question, it is necessary to first understand the role of TNFR2 in Teffs during a resolvable immune response. First, although TNFR2 stimulation in Teffs correlates with a high proliferative capacity and a high capacity to produce effector cytokines, TNFR2-deficient mice still display normal T cell development (Erickson et al., 1994). In addition, the time of exposure to TNF-α strongly shapes the consequences of TNFR2 activation on Teffs. A comprehensive scheme for the role of TNF-α/TNFR2 in Teffs during the natural resolution of inflammation by Tregs can be proposed as follows (Chen and Oppenheim, 2011a):

1) In the steady state, the equilibrium between Treg and Teff activation preserves immune homeostasis (Figure 2A).
2) In the early stage of inflammation, activated Teffs up-regulate their TNFR2 expression under TNF-α exposure, increasing their capacity to resist Treg-mediated inhibition, and therefore mount an effective immune response. In addition, a slower Treg response than that of Teffs to TNF-α results in delayed immunosuppressive feedback (Figure 2B; Chen et al., 2013).
3) In the later stage of inflammation, chronic exposure to TNF-α leads to impaired production of effector cytokines, caused by the competition of Tregs with Teffs for the co-stimulatory action of TNF-α/TNFR2 (Figure 2B; Aspalter et al., 2003; Clark et al., 2005). Importantly, TNFR2 also appears to be necessary for the sensitivity of CD8^+^ T cells to AICD (Kim E. Y. et al., 2009). At the same time, Treg TNFR2-dependent stimulation enhances their suppressive activity, resulting in the resolution of inflammatory responses and restoration of immune homeostasis.

The pattern of TNFR2 expression suggest that TNF-α can induce a potent immune reaction through monocyte/macrophage activation and T cell co-stimulation. At the same time, its activation launches a delayed immune response controlled through its expression on immunosuppressive cells. In pathological settings, including cancer, this fine balance between TNFR2 dual functions is lost due to changes in the pattern of expression. During carcinogenesis, tumor cells induce multiple modifications in the soluble milieu, including that of TNF-α levels because of its production by TME-cells, such as infiltrating T cells or macrophages, and by the tumor itself (Figure 2C; Josephs et al., 2018). They also recruit immunosuppressive cells, which further upregulate their TNFR2 expression under TNF-α exposure. This disturbs the equilibrium of the TNF-α/TNFR2 expression pattern, presumably putting cancer development in the third situation already described, i.e., the impairment of Teff function due to chronic exposure of both Tregs and Teffs to TNF-α. Consistent with this assumption, it was shown that the enhanced protection against lymphoma in TNFR2^–/–^ mice results from the recruitment, activation, AICD resistance, and subsequent prolonged survival at the tumor site of memory TNFR2^–/–^ CD8^+^ T cell, specific to tumor-associated antigens (Kim J. et al., 2009). Overall, these data suggest a beneficial action of TNFR2 blockade on Teffs anti-tumoral efficacy under chronic TNF-α exposure, despite the co-stimulatory role of TNFR2 in transitory inflammation. However, sufficient data is still lacking to untangle what the real consequences on immunity would be when using a TNFR2 blockade strategy as a therapeutic tool.

### Activation vs. TNFR2 Blockade

A recent study conducted by a pharmaceutical company evaluated the activity of murine and human anti-TNFR2 antibodies for cancer therapy (Tam et al., 2019). They described the mechanism of action of a novel mouse TNFR2 antibody (Y9) that appears to act through Fc-dependent agonism on conventional T cells, responsible for its antitumor activity in pre-clinical mouse models. In the subcutaneous tumor models they used, with both hematological and carcinoma cell lines, they observed no significant Treg depletion or dysfunction. A second pharmaceutical group screened for TNFR2 binders, a subset of which were found to be agonists for the receptor, leading to NF-κB pathway signaling *in vitro* (Williams et al., 2016). Their TNFR2-specific agonists inhibited tumor growth, enhanced tumor infiltration by CD8^+^ T cells, and increased CD8^+^ T cell IFN-γ synthesis in CT26 syngeneic tumors. In light of our hypothesis in the previous subsection, these results are not inconsistent with the previous results obtained using a TNFR2 antagonist. Indeed, antitumor cells that benefit from TNFα/TNFR2 pathway signaling for their activation can be found in the TME. As cited, they are mainly macrophages and conventional T cells. In a milieu under constant TNF-α saturation, a strategy to target immunosuppressive actors by blocking TNFR2 is probably the better angle of approach because Teffs are susceptible to Treg exhaustion after long TNF-α exposure. However, what about a tumor context in which no TNFR2 signal is delivered because TNF-α is absent? Then, in theory, providing a TNFR2 signal to proinflammatory TME-cells, as well as Tregs, could permit, at least transiently, the triggering of a potent anti-tumor Teff response, resistant to Treg suppression, while preserving tolerance. For instance, TNFR2 agonism could benefit to infiltrating macrophages among TNFR2^+^ cells in the TME. In the above-mentioned studies of Williams et al. (2016) and Tam et al. (2019) such an effect was not observed. However, in a later study, TNFR2 expressed on macrophages was proven to be sufficient to mediate the antitumor effect of TNF-α, probably by the inhibition of tumor angiogenesis (Zhao et al., 2007). This observation may be another interesting aspect for TNFR2 agonism in cancer, although more data concerning TNFR2, and myeloid cells are still required. Another goal that appears to be potentially obtainable by both TNFR2 agonists and antagonists is the direct impact of the treatment on tumor cells. Although TNFR2 blockade can divert cancer cells from a survival pathway, agonist molecules may cause a domino effect that could sensitize malignant cells to TNFR1-induced cell death by depriving them of cIAP/TRAF (Siegmund et al., 2016).

Due to its wide-ranging functions, TNF-α is also responsible for divergent actions in the context of cancer through its two receptors (Montfort et al., 2019). Far from what was initially thought about its antitumor effect, a growing body of clinical data supports the concept that chronic inflammation promotes tumor development and progression. As a major proinflammatory cytokine, reports have shown TNF-α to be involved in all aspects of carcinogenesis, from cellular transformation to survival, proliferation, invasion, angiogenesis, and metastasis (Wang and Lin, 2008). Hence, in phase I and II clinical trials, two anti-TNF-α antibodies (infliximab and etanercept) achieved prolonged disease stabilization in patients with metastatic breast cancer, recurrent ovarian cancer, or immunotherapy-resistant or refractory renal cell carcinoma (Madhusudan et al., 2004, 2005; Harrison et al., 2007). In the specific situation in which carcinogenesis is proven to be inflammation-driven, including through the proinflammatory effect of TNF-α, Tregs have been demonstrated to have a paradoxical protective effect. Thus, in this case, a strategy to block TNFR2 could be counterproductive by inhibiting the anti-inflammatory function of Tregs. Conversely, the utilization of an agonist to preserve Tregs while activating Teffs and macrophages could be more appropriate. Many examples of tumors for which Tregs may be protective can be cited, including colorectal carcinoma, in which gut microbiota driven-inflammation aids tumor growth (Erdman et al., 2003, 2005; Gounaris et al., 2009; Whiteside, 2012).

## Therapeutic Perspectives

### Current State of Clinical-Grade Therapeutic Tools

From the point of view of clinical utility, TNFR2 modulation appears to be an attractive approach in reshaping modern cancer immunotherapy. Targeting TNFR2 in cancer patients now requires clinical grade mAbs or small molecules, agonists, or antagonists, designed to target human TNFR2. Defining the right tool to translate murine findings to patients is a promising but challenging task. The TNF-α/TNFR2 crystal structure is now available, revealing the specific binding between TNF-α and TNFR2 (Mukai et al., 2010). In virtual screening, a library of compounds has been examined to predict their binding poses and affinities (Shaikh et al., 2018). Compounds that resemble the binding pose to the native ligand with better binding affinity will be selected as agonist candidates, together with new blocking mAb candidates, for further research and development in the drug discovery pipeline. As previously mentioned, a few academic groups have also published advances in testing anti-TNFR2 molecules in the context of cancer:

• Torrey et al. (2017, 2018) have developed an antagonist candidate that has proven to be efficient in inhibiting Tregs from patients with ovarian cancer and those with Sézary syndrome.
• Tam et al. (2019) screened for anti-human and anti- mouse TNFR2 agonist antibodies. They reported an antitumor effect on several cancer types using the murine version and found corresponding activity of human agonist TNFR2 antibodies that could be used in patients.
• Encouraging unpublished results were also presented at the AACR annual meetings in 2020 and 2021, regarding advances in both agonists and antagonists targeting TNFR2 and tested in the context of cancer (Mårtensson et al., 2020; Chen et al., 2021).

Although the development of good-manufacturing practice reagents to modulate TNFR2 in patients appears to be on track, finding an efficient a way to prove that mouse results can be translated to strategies for patients is currently less straightforward. *In vitro* testing of anti-TNFR2 tools is challenging as modeling T cell exhaustion or mid-activation of the TCR, to mimic pathologic situations, is complex. Furthermore, most available information concerns isolated Tregs, i.e., mostly Tregs independent of TNF-α exposure. Finally, several groups are trying to assess TNFR2 modulation strategies in human cells *in vivo* using immunodeficient mice. He et al. (2016) were the first to publish preliminary results using a commercialized TNFR2 agonist in a humanized skin allograft model. Tam et al. (2019) tested their agonist candidate in a humanized model of colorectal adenocarcinoma without success. Using patient-derived xenografts, this group then observed an antitumor effect upon TNFR2 activation when combined with an anti-PD-1 relative to PD-1 monotherapy. No TNFR2 inhibition studies in humanized mouse models have yet been published. As for *in vitro* models, testing new immunotherapies using humanized mouse models is challenging, in part because xenogeneic models trigger excessive T cell activation, which does not reflect clinical reality in cancer (Scalea et al., 2012). Further *in vitro* and *in vivo* studies are also needed to better define the intracellular events that follow agonist or antagonist candidate binding to human Tregs and Teffs, as well as to other types of TNFR2-expressing cells to envisage potential off-target effects.

### Potential Side Effects

Anti-CTLA-4 and anti-PD-1/PDL-1 mAbs have shown high antitumor efficacy in responding patients. However, a major drawback associated with the use of ICIs agents is the apparition of severe autoimmunity/autoinflammatory symptoms (Young et al., 2018). These therapies are able to trigger or restore patients’ effector responses against tumor cells, which express both tumor-associated antigens and self-antigens (Shimizu et al., 1999). The subsequent over-activation of Teffs against self can lead to diverse organ lesions. In addition, these antibodies can induce deleterious autoimmune effects by binding off-target, for example, diabetes mellitus after PD-L1 treatment due to PD-L1 expression by pancreatic β-cells (Fousteri, 2020). Because such chronic effects deeply affect the patients’ quality of life, treatment-safety is a major consideration for the new generation of immunotherapies to come.

One important feature of TNFR2 is its restricted perimeter of expression, raising the hope of limited side effects under therapeutic utilization, contrary to TNF-α or TNFR1 targeting. Because activated T cells upregulate TNFR2, a potential safety concern comes from its inducible expression on Teffs upon TCR stimulation. In addition, co-stimulation through TNFR2 on Teffs could improve their ability to resist Treg-mediated suppression in tumors. Nevertheless, as Tregs in the TME appear to persistently express higher levels of TNFR2 than Teffs, the assumption that this treatment should have a more profound impact on Tregs than Teffs appears to be plausible. Thus, the net outcome of TNFR2 antagonism could favor Teff activation and expansion, triggering the establishment of an effective antitumor immune response. This hypothesis will require further testing, with careful assessment of the impact on the effector response at each step following TNFR2 inhibition, as well as that on pro-inflammatory TNFR2^+^ myeloid cells, which could be impaired by a TNFR2-blockade strategy. Importantly, apart from the immune compartment, TNFR2 is expressed and upregulated in pathological situations, such as ischemia and in endothelial cells and neural tissue at the surface of local macrophages (microglia), as well as other non-neuronal cells (astrocytes) (Fischer et al., 2020). Whether TNFR2 blockade would have off-target effects in these two tissues or non-desired immune effects through myeloid cells will need to be carefully monitored.

In our hands, a blocking mAb against TNFR2 induced a change in phenotype of Tregs when used in a model of allogeneic hematopoietic stem cell transplantation and presumably impaired their suppressive capacity but did not deplete them (Leclerc et al., 2016). The preservation of a viable Treg pool, although less functionally potent, could better prevent autoimmunity than complete Treg depletion. Our observations are consistent with the fact that TNFR2^–/–^ mice do not develop autoimmunity, suggesting that the restriction of TNFR2 on a Treg subset in the steady-state does not impair the capacity of other subpopulations to maintain an immune balance (Vanamee and Faustman, 2017). In cancer, an advantage of TNFR2 is its higher expression among certain tumor-infiltrated Tregs than Tregs in the circulation. The dominant TNFR2 antagonists from the study of Torrey et al. (2017) preferentially suppressed the activity of tumor-associated Tregs but had no or only minor inhibitory effects on regular Tregs in the periphery, which play a crucial role in the maintenance of immunological homeostasis. Importantly, no evidence has been yet reported concerning the transient or long-term nature of the effect observed on Tregs after TNFR2 blockade.

### Hopes for Combined Strategies

The clinical use of ICIs has shown limitations in terms of the frequency of responding patients. Anti-PD-1 combinations with chemotherapy or other immunotherapies, such as anti-CTLA-4, have been able to improve efficacy, but often at the expense of substantial increases in toxicity relative to anti-PD-1 alone (Weber et al., 2016; Paz-Ares et al., 2018). As Tregs could be a brake that limits the action of these molecules, the depletion of Tregs or reduction of their suppressive activity are two strategies that could enhance currently available treatments. Tumor killing using chemotherapeutic drugs, irradiation, or ICIs may release self-antigens and tumor-associated antigens and cause local inflammation, possibly resulting in the recruitment of Tregs to tumor tissues, their activation, then hampering ensuing antitumor immune responses. Thus, an anti-Treg strategy could be used with pro-Teff immunotherapies to strongly activate Teffs while avoiding interference from Treg recruitment.

If combinational strategies could improve the therapeutic efficacy of a single agent, they could also possibly enlarge the range of indications compared to monotherapies. Combined approaches are being designed to address the issue of non-infiltrated/cold tumors, for which immunotherapy alone is inefficient (Bonaventura et al., 2019), for instance to normalize vascularization, allowing a better antitumor cells infiltration. In this setting, TNFR2 is a highly interesting candidate since its expression on endothelial cells and immunosuppressive cells makes it essential for both chaotic tumor angiogenesis (Sasi et al., 2012) and the maintenance of an immunosuppressive TME. For these reasons, TNFR2 blockade used in combination with an angiogenesis regulator could permit both the regulation of tumor vascularization, allowing an effective Teff infiltration and a more potent antitumor response.

## Concluding Remarks

Immunotherapy is currently sparking an extraordinary level of energy and enthusiasm among the scientific community and healthcare industry due to its potential for the treatment of solid and hematological malignancies. A better understanding of Treg dysregulation in cancers during the last two decades has opened a new therapeutic window in the field. Clinical strategies to specifically inhibit Tregs without affecting Teffs are still challenging, largely because of phenotypic similarities shared between the two cell subsets. A myriad of tools will undoubtedly be available in the near future, making it possible to increasingly consider the patient’s cancer type and immune status. TNFR2 is a versatile target option, of which both activation and blockade could serve as an anti-cancer therapy, probably for distinct therapeutic situations. It is important to stress the inclusion of such an approach in the concept of personalized medicine. Based on our current knowledge, the use of anti-TNFR2 in therapy will have to be context-dependent, with TNF-α levels in the tumor surroundings and the time of exposure to this cytokine (depending on the tumor type and disease stage) being potentially the most crucial factors to consider when using such agents. Future pre-clinical experiments should probably focus on comparing agonist and antagonist effects in the same model to determine whether the previously discussed assumptions are accurate. Importantly, TNFR2 is not the only target-molecule to offer such a possibility for dual modulation. An entire class of new therapeutics focuses on costimulatory molecules preferentially expressed on Tregs, such as GITR, ICOS, OX40, 4-1BB, and DR3, creating a new potential drug class of “checkpoint stimulators,” completing the current available ICI options and allowing new combinations of immunotherapies (Chen and Oppenheim, 2017).

Because of the high toxicity associated with the ICIs currently used in the clinic, there is an ongoing need for new cancer immunotherapies that show promising activity but are also well tolerated. Targeting TNFR2 in monotherapy could spare a functional subset of peripheral Tregs, which offers hope about its capacity to be well tolerated. Nevertheless, the risk of autoimmunity when blocking TNFR2 is considerable, as for other new ICIs candidates, and difficult to evaluate outside of proper clinical trials. The availability of an antagonist and agonist of clinical relevance makes it possible to envisage shortly launching clinical trials in cancer patients. However, the type of tumor to include in these assessments is yet to be precisely determined. Compelling results on patient-derived Tregs are already available for a human TNFR2 antagonist. The presence of TNFR2^+^ Tregs has also been reported in numerous other types of malignancies, which will help in choosing the first cohorts.

## Author Contributions

AM wrote the first draft of the manuscript. Both authors wrote together the final version of the manuscript.

## Conflict of Interest

The authors declare that the research was conducted in the absence of any commercial or financial relationships that could be construed as a potential conflict of interest.

## Publisher’s Note

All claims expressed in this article are solely those of the authors and do not necessarily represent those of their affiliated organizations, or those of the publisher, the editors and the reviewers. Any product that may be evaluated in this article, or claim that may be made by its manufacturer, is not guaranteed or endorsed by the publisher.
